# Wireless signal modulation identification method based on RF I/Q data distribution

**DOI:** 10.1038/s41598-021-00723-7

**Published:** 2021-11-01

**Authors:** Lihui Wang, Zhenjia Chen, Yonghui Zhang

**Affiliations:** School of Information and Communication Engineering, Hainan University, Haikou, 570228 China

**Keywords:** Computational platforms and environments, Data processing

## Abstract

Electromagnetic spectrum detection is the basis of the next generation wireless communication technology. Wireless signal identification is an important part of electromagnetic spectrum detection and management activities. This paper proposes to extract the distribution features of different modulated signals from the signal I/Q data. A two-dimensional gradient matrix is used to describe the characteristics of the signal classification. The minimum gradient cumulative distance (GCD) estimate between the sample and the model is used as the decision criterion for the signal classification. According to the result of the confusion matrix, the weight of the model is adjusted. Experiments show that the recognition rate of the modulated signal mentioned in this paper can reach 82.75%. The I/Q data sample was extracted under actual engineering conditions involving random noise, and the recognition rate dropped to approximately 79%. Based on the initial model gradient matrix, a reasonable algorithm is set to adjust the weight of the model, which can effectively improve the recognition rate of the modulated signal.

## Introduction

Electromagnetic spectrum resources are non-renewable resources. The use of spectrum resources by various countries is an important national development strategy. The electromagnetic spectrum detection system mainly realizes the perception and visualization of the spatial spectrum distribution. This is the basis for the safe and rational use of spectrum resources from the physical layer. In the spectrum detection system, most researchers pay attention to the spatial spectrum distribution prediction algorithm, spectrum occupancy calculation, and spectrum interference detection algorithm^1^. The study of interference maps helps manage spectrum resource utilization, facilitates the rapid location of interference sources, and develops solutions^2,3^. However, these spectrum detection methods only estimate the energy distribution of the spatial spectrum and do not estimate other signal characteristics. As the number of wireless devices increases, analyzing electromagnetic spectrum detection from the perspective of source feature extraction can more effectively improve the electromagnetic spectrum resource management and control capabilities. For example, the location, tracking and monitoring of unregistered signals. Describe the spectral resources based on the signal source parameters that can greatly reduce the capacity of the spectrum database^4^. Therefore, the research of signal detection methods directly affects the accuracy of spectrum detection results. Researchers propose an algorithm for signal identification problems specific to MIMO systems^5^. Among them, the researchers conducted in-depth research on space-time block code identification and MIMO modulation identification. The MIMO modulation identification methods are mainly divided into: the maximum likelihood function method of the received signal, and the specific modulation features of the received signal. The data processed by the system is obtained after the Fast Fourier transform (FFT) block of the I/Q data at the Radio Frequency (RF) end.

**Figure 1:**
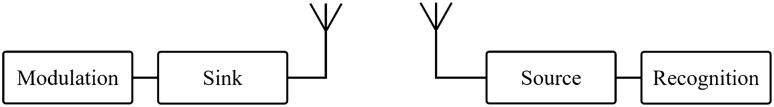
Wireless signal transmission and reception. At the transmitting end, the software radio module transmits the modulated signal through the *Sink* to transmit I/Q data to the antenna end. At the receiving end, the software radio module obtains the I/Q data of the antenna end from the *Source*.

Blind detection is a necessary condition for the spectrum detection system. Matching detection of a specific modulation method increases the complexity of the detection system. With the digitization of wireless signals, we can achieve signal source identification through data analysis methods. RF I/Q data contains all the information of the wireless signal source. We extract the phase information at different receiving antennas from the I/Q data to achieve signal source direction finding^6^. The software defined radio (SDR) module collects I/Q data from the source. FFT is performed on the I/Q data to obtain the energy distribution of the signal at each frequency. Real-time phase information can also be extracted from the I/Q data. By extracting real-time phase information from the I/Q data, we implemented low signal to noise ratio (SNR) signal detection^7^. The data after FFT ignores many features. Therefore^8^, establishes a convolutional neural network (CNN) based on three features of I/Q, Amplitude/Phase and FFT to realize the recognition of signal modulation. But the authors use the I/Q data before the RF end after the modulation module as shown in Fig. 1. Different modulation signals can be easily distinguished according to the I/Q distribution. However, it is not suitable for practical engineering applications.

**Figure 2:**
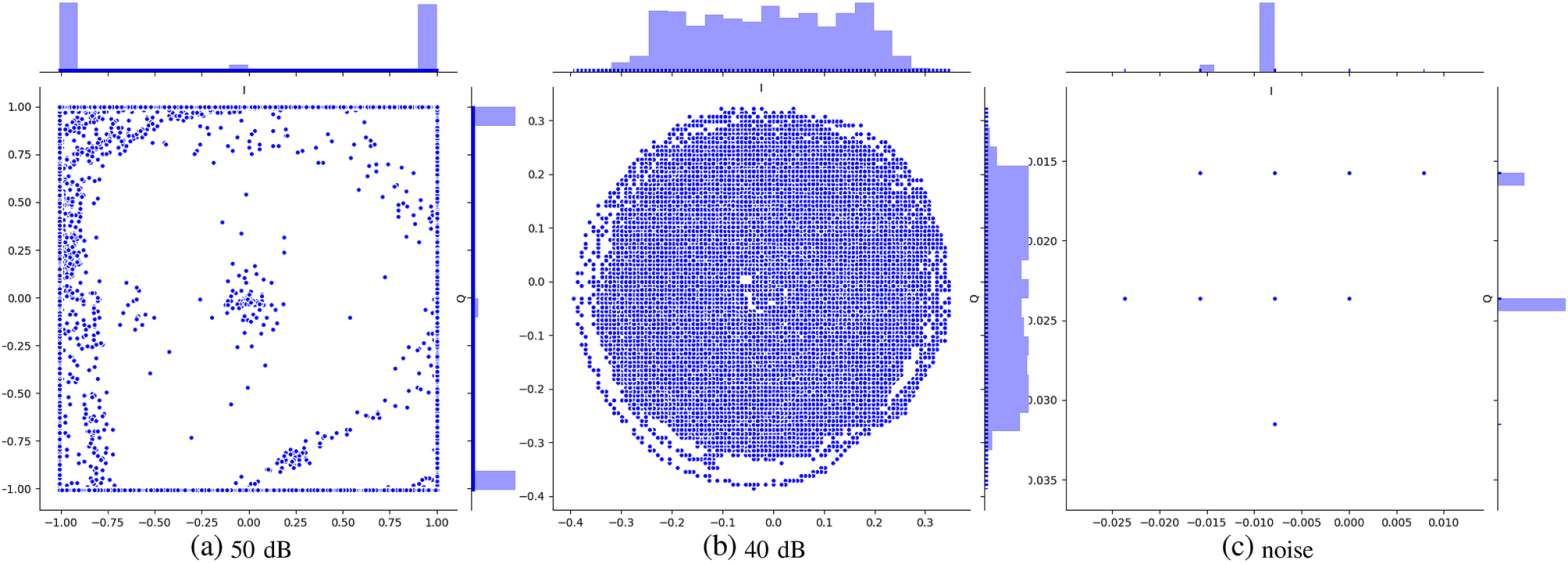
The distribution of I/Q data components of radio signals in environments with different SNR (one I/Q data sample). The higher the signal strength, most of the components of the I/Q data are concentrated in the positions of the four vertices of the coordinates. The weaker the signal strength, the I/Q component gradually approaches the origin.

The main contribution of this paper is to study the application of I/Q distribution characteristics in modulation signal recognition. The received I/Q data contain the noise of the environment, and its I/Q distribution will change accordingly. The I/Q distribution of signals with different modulation modes has different distribution features. Compared with Amplitude/Phase and the output FFT, I/Q data can better reflect the features of the signal. We extract the I/Q data of the wireless signal source directly from the source module at the receiving end. The I/Q data is the signal after the frequency spectrum is moved to the baseband. It contains the original information of environmental noise. Neural networks can be applied to classification, but learning time is too long, it is difficult to explain the reasoning process and reasoning basis. If the model adds a new category, it will need to be re-learned and will affect the recognition accuracy of the old category. The amount of I/Q data sample is large, and the time it takes to learn with a convolutional neural network is greatly increased. We propose to implement wireless signal modulation recognition based on I/Q component probability distribution.

## I/Q component distribution of different modulation signal source

The SDR collects wireless signals in the form of I/Q data. Due to a large amount of I/Q data, the frequency distribution of the signal can be easily analyzed by frequency domain analysis after FFT. The time-frequency domain spectrum distribution characteristics can classify the signal modulation. However, the energy detection method usually has a high probability of missed detection ($P_{m}$) at low SNR. In fact, the I/Q data obtained by the RF receiver will be mixed with noise, and its I/Q distribution is distributed in different forms of “ring”. We propose to describe the signal features by extracting the I/Q distribution of different modulated signals. The I/Q data contains all the features of the baseband signal. I/Q data appears in complex form. It can be expressed as

1 $$\begin{aligned} S(n) = I + jQ, \quad I,Q \in [-1, 1]. \end{aligned}$$

In this paper, signal samples with different energy intensities are collected the I/Q component distribution of different SNR signals under the condition of no environmental interference is shown in Fig. 2. I represents the abscissa, Q is the ordinate, and the histogram represents the real part and the distribution component. When the signal strength is strong, most of the I/Q components are distributed near the four vertices of the interval, and there are fewer I/Q components near the origin, as shown in Fig. 2a. With the signal strength decreases, the I/Q signal gradually converges to the origin, the number of I/Q components decreases, and the real and imaginary parts are approximately normally distributed, as shown in Fig. 2b. However, the number of I/Q components of the noise sample is close to zero, as shown in Fig. 2c.

**Figure 3:**
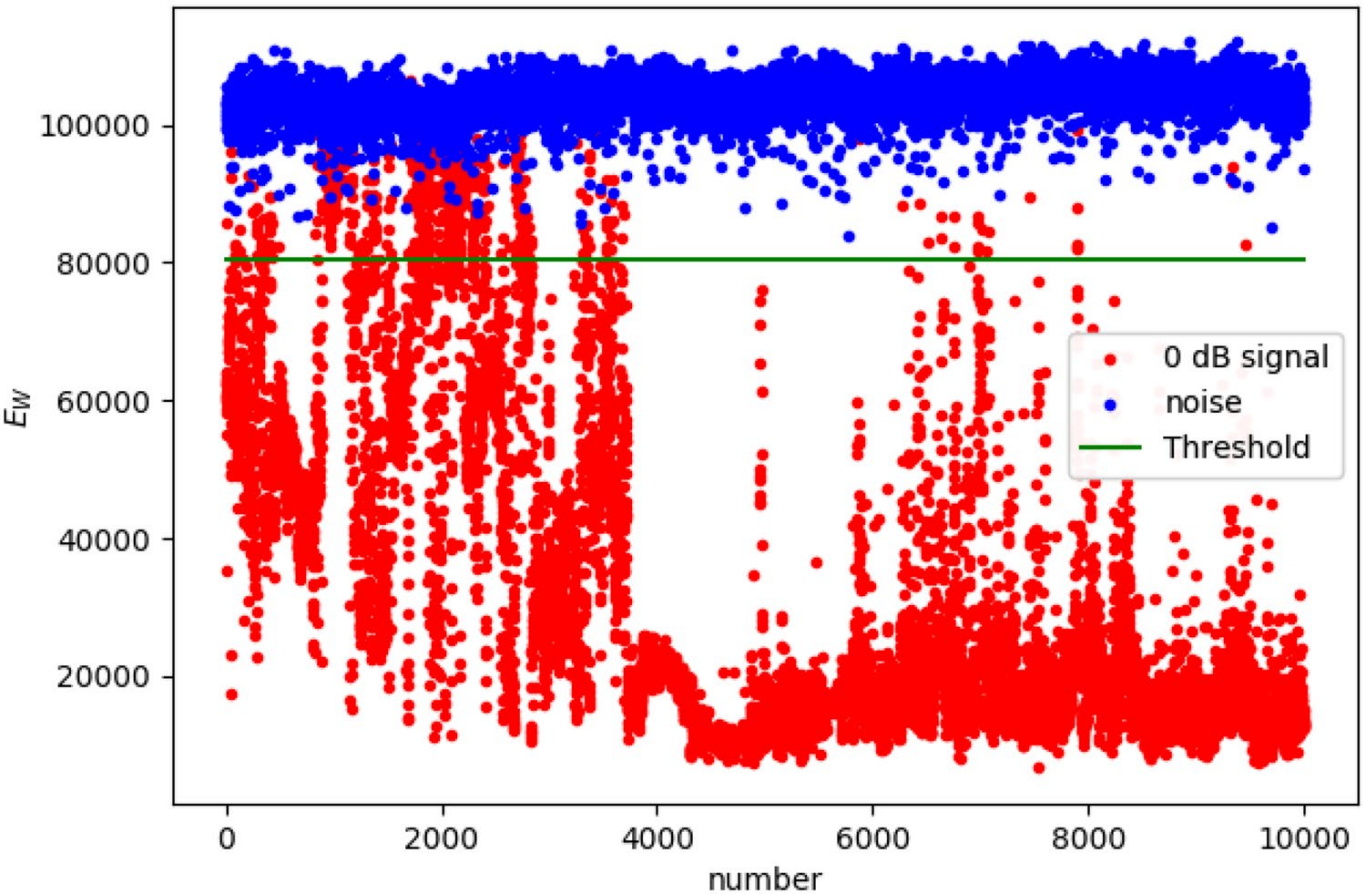
Weak signal detection by I/Q data weight extremes distribution method. The X-axis coordinate is expressed as $E_{W}$ of an I/Q sample. The $E_{W}$ of the noise is concentrated on a relatively high value.

The amount of I/Q for each sample of SDR acquisition is primarily determined by the ADC/DAC performance of the SDR. The sampling number of the software radio module used in this article is 8 bit, and the sampling rate is 20 Msps. The I/Q distribution is described by the real part and imaginary part of the complex number, and the Z-axis coordinates describe the weight of the I/Q component. In our detection system, the amount of I/Q for one samples is $n \in [1, 1,131,072]$. The component number of the sample is defined as *cp*. The weight of an I/Q component is defined as $C^{w}_{i,j} = Count(C^{real}_{i} = I, C^{imag}_{j} = Q)$. In the case of low SNR, 10,000 weak signal samples and 10,000 noise samples are collected, and the $E_{W}=max(C^{w}_{i,j})$ distribution of the samples is shown in Fig. 3. As the SNR decreases, the I/Q component approaches the origin, and the weight of the largest I/Q component can reach $C^{w}_{i,j} \rightarrow 100,000$. When the boundary between noise and signal is set to $E_{W}=80,000$, the detection probability of weak signals can reach more than 95%. As the SNR increases, the I/Q component is concentrated toward the four coordinate boundary points $(-1, -1)$, $(-1, 1)$, $(1, -1)$, (1, 1). Moreover, under the same SNR, the I/Q distribution in the complex coordinates of different modulated signals is also different. After the I/Q data is statistically calculated in the same real part and imaginary part, I/Q component distribution can be expressed as

2 $$\begin{aligned} \{C^{real}_{i}, C^{imag}_{j}, C^{w}_{i,j}|i,j=1, \ldots ,cp\}, \end{aligned}$$

where $C^{real}_{i} = I$ and $C^{imag}_{j} = Q$. The positions of $C^{real}_{i}$ and $C^{imag}_{j}$ in the coordinates reflect the spatial distribution of the I/Q signal.

## Modulation identification method base on I/Q distribution

**Figure 4:**
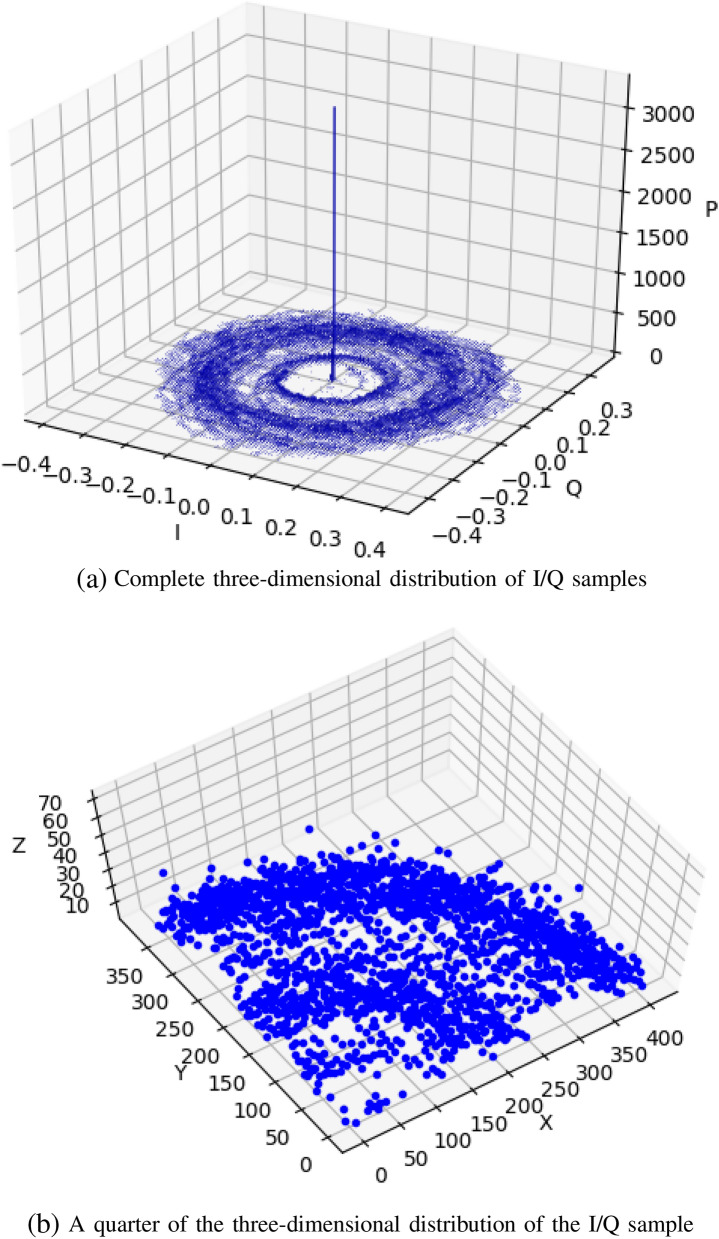
I/Q distribution of FSK signal (40 dB). I/Q components are mainly distributed in $I,Q \in [-0.4, +0.4]$.

With the same SNR, the I/Q component distribution of the wireless signal has a similar pattern. We propose a model that combines the gradients of the I/Q distribution to describe the different modulation signals. As shown in Fig. 4a, it is the distribution of I/Q samples of a complete FSK signal. The I/Q sample distribution of the modulated signal after propagation in free space will show the characteristic of “ring”. According to the gradient distance between the sample and the model, the model with the smallest distance is the target category. In order to reduce the amount of data, we take the data of $I,Q \in [0, 1]$. The data volume of the model is one quarter of one I/Q data sample, as shown in Fig. 4b. The real part *I* of each signal *S*(*n*) is used as the X-axis coordinate, and the imaginary part *Q* is used as the Y-axis. The number of multiple *S*(*n*) is the value of the Z-axis coordinate, and finally reflects the I/Q sample distribution of the modulated signal. The modulation signal identification method proposed in this paper is mainly related to the SNR of the signal. The lower the SNR of the signal, the more concentrated the distribution of the I/Q component is at the zero point. The I/Q samples used in this article are all the SNR of the modulated signal is 40 dB.

If the real part and imaginary part of I/Q are accurate to 0.001($I,Q \in [0.000, 0.999]$), the matrix of $1000 \times 1000$ represents the I/Q component distribution. The two-dimensional I/Q distribution matrix can be modified to

3 $$\begin{aligned}C^{w \; 1000 \times 1000} = \\&\begin{bmatrix} C^{w}_{0, 0} &{} C^{w}_{0, 1} &{} ... &{} C^{w}_{0, j} &{} ... &{} C^{w}_{0, 999} \\ C^{w}_{1, 0} &{} C^{w}_{1, 1} &{} ... &{} C^{w}_{1, j} &{} ... &{} C^{w}_{1, 999} \\ \vdots &{} \vdots &{} &{} \vdots &{} &{} \vdots \\ C^{w}_{i, 0} &{} C^{w}_{i, 1} &{} ... &{} C^{w}_{i, j} &{} ... &{} C^{w}_{i, 999} \\ \vdots &{} \vdots &{} &{} \vdots &{} &{} \vdots \\ C^{w}_{999, 0} &{} C^{w}_{999, 1} &{} ... &{} C^{w}_{999, j} &{} ... &{} C^{w}_{999, 999} \end{bmatrix}\end{aligned}$$

where $C^{w}_{i,j}$ can describe the value of the I/Q component in different SNR environments. When it is a noise sample, then $C^{w}_{i,j} > 80,000$. When it is a strong signal, there is $C^{w}_{i,j} \rightarrow 1$ except for (1, 1). So $C^{w}_{i,j} \in [1, 80,000]$. $C^{w \; 1000 \times 1000}$ can represent I/Q data samples in different SNR environments.

We propose to describe different modulation signals by extracting a two-dimensional gradient matrix from the two-dimensional I/Q distribution matrix. The two-dimensional gradient matrix is obtained by calculating the gradient of adjacent $C^{w}_{i,j}$ in the two-dimensional I/Q distribution matrix. Two-dimensional gradient matrix as the basic model for adjusting signal recognition. It can be expressed as

4 $$\begin{aligned} G^{w}_{i,j}&= \left\{ \begin{matrix} C^{w}_{i + 1,j} - C^{w}_{i,j} \\ C^{w}_{i,j + 1} - C^{w}_{i,j}, \end{matrix}\right. \end{aligned}$$

where the first equation is the column gradient, and the second equation is the row gradient. The two-dimensional gradient matrix model of this paper is calculated by column gradient. The two-dimensional gradient matrix formula of the column gradient is shown in (5).

5 $$\begin{aligned}G^{w \; 1000 \times 1000} = \\&\begin{bmatrix} C^{w}_{1,0} - C^{w}_{0,0} &{} ... &{} C^{w}_{1,j} - C^{w}_{0,j} &{} ... &{} C^{w}_{1,999} - C^{w}_{0,999} \\ C^{w}_{2,0} - C^{w}_{1,0} &{} ... &{} C^{w}_{2,j} - C^{w}_{1,j} &{} ... &{} C^{w}_{2,999} - C^{w}_{1,999} \\ \vdots &{} &{} \vdots &{} &{} \vdots \\ C^{w}_{i + 1,0} - C^{w}_{i,0} &{} ... &{} C^{w}_{i + 1,j} - C^{w}_{i,j} &{} ... &{} C^{w}_{i + 1,999} - C^{w}_{i,999} \\ \vdots &{} &{} \vdots &{} &{} \vdots \\ 0 &{} ... &{} 0 &{} ... &{} 0. \end{bmatrix} \end{aligned}$$

For the same modulated signal, the two-dimensional I/Q distribution matrix between samples will have a small change, and the corresponding two-dimensional gradient matrix will also have a difference. In the case of the same SNR, we propose GCD to identify the modulated signal. It can be expressed as

6 $$\begin{aligned} D_{GCD}&= \sum \left| G^{w}_{label} - g^{w} \right| \\&= \sum _{i=0,j=0}^{x\_max-1,y\_max-1} \left| G^{w}_{i,j} - g^{w}_{i,j} \right| . \end{aligned}$$

In practical applications, random noise has an influence on the recognition rate of the modulated signal. We propose model weight parameters (*W*) to adjust the gradient distance of each category and improve the overall recognition rate. (6) can be modified as

7 $$\begin{aligned} D'_{GCD} = \sum \left| W_{label} \times G^{w}_{label} - g^{w} \right| . \end{aligned}$$

The value of *W* depends on the modulation signal identification error detection result. The similarity of the models of the individual categories is higher, and the GCD is lower than the actual value due to random noise, resulting in a error detection. The initialization model is a single sample gradient matrix, and the self-feedback model weight parameter adjustment can effectively improve the overall recognition rate.

## Results and analysis

**Figure 5:**
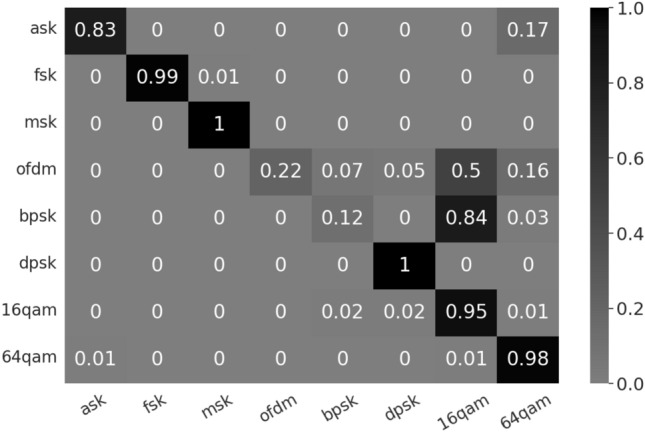
Confusion matrix for the wireless signal modulation identification (Before adjusting parameters). In the confusion matrix, each column represents the predicted category, and each row represents the true belonging category of the data.

The signal acquisition module is HackRF. Its ADC/DAC sampling rate can reach 8 bits, that is, the range of I/Q component distribution is 1–256. A single transmitting antenna transmits signals, and a single receiving antenna collects I/Q data at a distance of 20 cm. The transmit power of the signal source is directly measured as − 70.07 dBm with the feeder. The SNR of samples is about 40 dB. Based on the open source energy detection project ($soapy\_power$), we modify the script to calculate the weight of the I/Q sample data to obtain a two-dimensional I/Q distribution matrix. The size of a single initial two-dimensional gradient matrix model is only 8.0 MB^9^. It can be deployed directly to the spectrum detection system. Confusion matrix for the wireless signal modulation identification as shown in Fig. 5. There are a total of eight types of modulated signals in this paper^10^, which are collected and saved as I/Q data formats by the SDR module. There are 100 test samples for each type of signal (800 samples in total). The recognition rate of the modulated signal can reach 76.13%.

**Figure 6:**
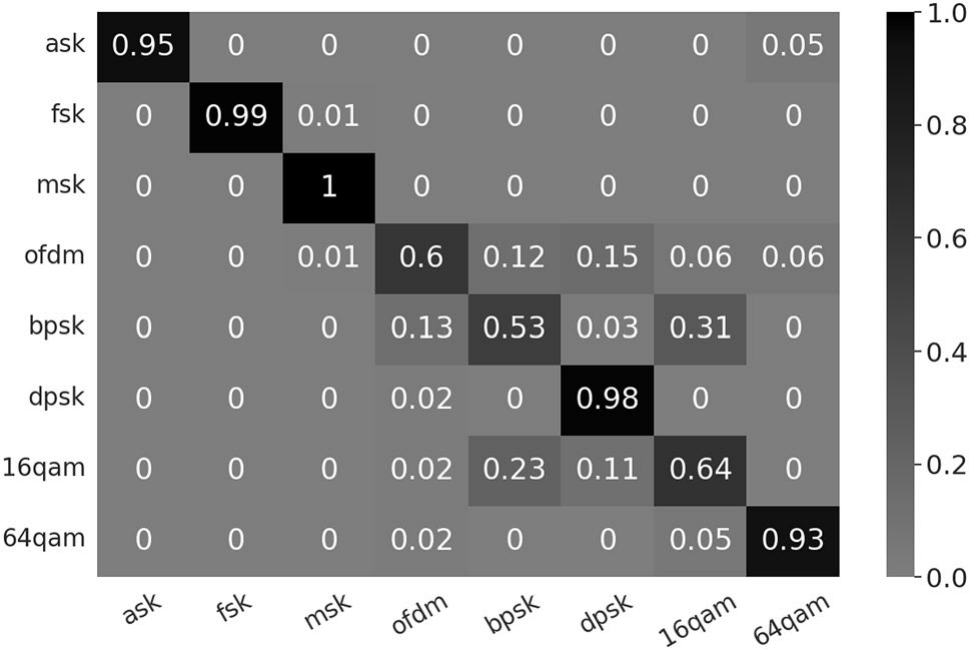
Confusion matrix for the wireless signal modulation identification (after adjusting parameters).

In current newer research, the modulated signal I/Q data sample after modulation module and before RF module is used^8^. The sample in this article is the I/Q data sample from the RF module. The signal transmission and reception module introduces random noise. Even so, the recognition rate of the GCD method can also approach the recognition rate of the^8^ method. I/Q distributions are all distributed in a “ring” shape. OFDM, BPSK and 16QAM have similar I/Q distributions. Since most samples of OFDM and BPSK were misidentified as 16QAM, the recognition rate of OFDM and BPSK is low. According to the confusion matrix, it can be found that the probability of being misjudged as 16QAM and 64QAM is high, and the overall recognition rate can be improved by increasing their GCD coefficients. In order to improve the recognition rate, the model weight is adjusted. Suppose the initial model weight vector is [1, 1, 1, 1, 1, 1, 1, 1], and the model weight step value is 0.01. As shown in Fig. 5, the samples of OFDM and BPSK are mostly misclassified as 16QAM and 64QAM. Therefore, it is preferred to increase the model weights of 16QAM and 64QAM step by step to increase the relative threshold of GCD and the recognition rate of OFDM and BPSK. In the adjustment process, the model weight of a single category model is increased to ensure that the overall recognition improves until the overall recognition rate converges. The model weight vector after recognition rate convergence is $D'_{GCD}=[1,1,1.05,1,1,1,1.05,1.05]$. The recognition rate has increased to 82.75%. Confusion matrix for the wireless signal modulation identification after adjusting parameters as shown in Fig. 6. After adjusting the model parameters, the recognition rate of 16QAM is reduced, and the recognition rates of OFDM and BPSK are improved.

**Figure 7:**
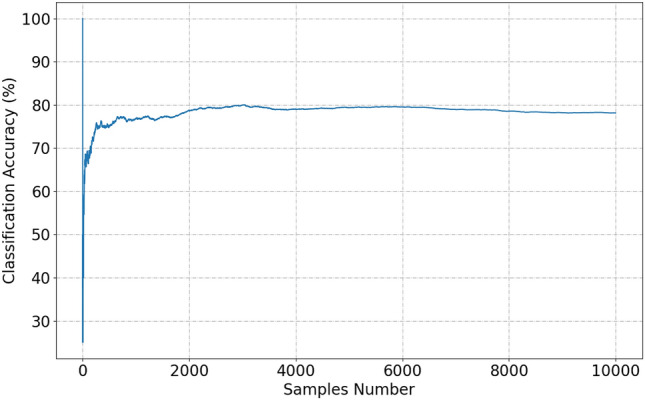
Classification accuracy in a large sample size experiment. The modulation signal of each experiment is randomly selected.

We mainly adjust the weight manually, so that the weight of the modulation signal with higher similarity is reduced, and the weight of the adjustment signal with less obvious characteristics is increased. The I/Q data sample used in this paper is measured data and environmental noise has been introduced. In different SNR environments, the I/Q distribution of the modulation signal does not change much, but the weights need to be adjusted slightly. Finally, we conducted engineering tests. Each sample is randomly selected to calculate the recognition rate. After a large sample size experiment, the recognition rate of actual engineering tests converges to 79%. As shown in Fig. 7. In the future, we will study the dynamic model weight adjustment method based on error detection results to improve the recognition rate.

## Conclusion

We propose a modulation signal identification based on the signal I/Q distribution and the two-dimensional gradient matrix by estimating the gradient cumulative distance. The I/Q distribution matrix is extracted with a single I/Q data sample and the gradient matrix is calculated. The gradient distance of the sample describes the degree of similarity of the model. Then, the recognition rate of the modulated signal is increased by the model weight. Under the influence of random noise, the measured results show that the proposed method can guarantee a higher recognition rate.
